# Reflections on physiology and modelling

**DOI:** 10.1113/EP091711

**Published:** 2024-01-15

**Authors:** Michael J. Joyner

**Affiliations:** ^1^ Department of Anesthesiology and Perioperative Medicine Mayo Clinic Rochester Minnesota USA

**Keywords:** blood pressure, exercise, peripheral circulation

## 1 INTRODUCTION

In this issue of *Experimental Physiology*, Furst and González‐Alonso detail an unconventional or alternative model of the circulation (Furst & González‐Alonso, 2024). Their model challenges the centrality of the heart as a pressure‐generating pump and the cardiocentric perspective that dominates what might be called the standard model of the circulation. The main idea behind this alternative model is that the metabolic demands of the tissue drive cardiac output, with the heart coordinating or causing ‘dynamic equilibrium’ between the arterial and venous arms of the circulation. As the authors point out, their model is not the first time a non‐cardiocentric or less cardiocentric model has been proposed. They also note an impressive historical list of eminent physiologists who have, with some controversy, challenged the cardiocentric perspective with at least some focus on the role of the venous system in cardiovascular regulation (Brengelmann, 2019). I might also add that my teacher John Shepherd told me repeatedly that he felt that the veins and regulation of the venous system, especially in the context of the upright posture in humans, had not received enough attention (Joyner, 2011).

There are many outstanding points in the model detailed by Furst and González‐Alonso, and hopefully the paper will generate vigorous debate and new experiments. But let us first consider whether the dominant model is really cardiocentric and then how and why the cardiocentric model became so dominant. Then let us consider a larger question: why make models?

## 2 IS THE DOMINANT MODEL OF CIRCULATION CARDIOCENTRIC?

I would argue that the dominant model is not highly cardiocentric but is arterial blood pressure centric. In this model, as exemplified by the ‘Gore diagram’ I learned as a student, blood pressure is a regulated variable sensed on a beat‐by‐beat basis by the arterial baroreflexes, with longer‐term regulation by the kidney (Joyner et al., 2011). The model is thus a hydraulic model, but it is also a feedback control model. An acute loss of reflex control of blood pressure for more than a few seconds leads to hypotension, fainting and loss of consciousness, providing a dramatic example of perhaps why the hydraulic/control model is so attractive.

On both a qualitative and a quantitative basis, the hydraulic/control model can be used to understand how blood pressure and blood flow change and how they are regulated in different physiological conditions. It can also be used to understand how pathological conditions affect one or more elements of the system. Additionally, each of the main elements of the Gore diagram is a site for therapeutic intervention. If you understand it, you understand a lot about drug targets for diseases such as hypertension and congestive heart failure.

When I was a medical student at the University of Arizona in the early 1980s, we did a laboratory exercise using a simple mechanical model of the heart. It had a piston pump that could be adjusted to simulate changes in stroke volume. Heart rate could be altered by changing how many times the piston went up and down per minute. The filling pressure of the heart could be increased or decreased via a reservoir. The vascular resistance and organ blood flows could be controlled by a series of taps. Students were thus the controllers who manipulated various elements of the system to see what impact they had on cardiac output and mean arterial pressure. As I recall, the only thing missing was something that simulated renal regulation of fluid balance, which contributes importantly to long‐term blood pressure regulation, via diuresis when arterial pressure is too ‘high’ and fluid retention when arterial pressure is too ‘low’. The model was fun and a simple but impressive teaching tool that reinforced key concepts in the Gore diagram. It also helped that Professor Robert Gore was one of the physiology teachers supervising our laboratory experience.

## 3 WHERE DO MODELS COME FROM?

Before the industrial revolution, in 1628 William Harvey described the fundamental principles of the circulation with the heart as a pump. Over the next 300 years or so, more knowledge about the peripheral circulation and the regulation of blood pressure accumulated. During the same time period, physicists and engineers developed data‐driven ideas about fluids flowing in pipes, hydrology, and control systems for industrial machines and processes. The engineering parallels with the circulation are obvious. Thus, it does not take a lot of imagination to see how a model emerged with the heart as a pump and a series of resistors that distribute blood flow to various organs under both feedforward and feedback control of the autonomic nervous system and kidney. Based on that perspective, I would argue that much of the progress in cardiovascular physiology has happened because physiologists creatively and productively borrowed concepts from or at least exchanged them with the physicists and engineers. Something similar has happened recently, as fundamental ideas about information theory have been useful to geneticists who think about DNA in the context of information transfer and computer coding (Jost, 2020). Others, such as Denis Noble, have argued that a keyboard and musical note analogy can be useful when thinking about genetics (Noble, 2008).

It is also important to remember that when it comes to the heart, the non‐scientific mythology of the heart as something akin to the seat of the soul made it an attractive candidate to be at the centre of any model of the circulation. Additionally, the isolated, perfused, beating heart preparations pioneered by Langendorff and Starling were used to generate foundational knowledge of cardiovascular physiology (Bell et al., 2011; Knowlton & Starling, 1912). Anyone who has ever seen such a preparation and done fundamental things, such as changing the filling pressure or adding catecholamines to the perfusate, can only come away with personal ‘aha’ moments that make a deep impression.

## 4 THE VALUE OF MODELS IN PHYSIOLOGY?

Models are useful to help us integrate our thinking about complex systems. They can be used to make predictions. They can also be useful to help us understand what we do not know and where there are gaps in knowledge. When gaps in knowledge are identified, new questions and intellectual opportunities emerge. When something does not ‘fit’ the model, the model has done one of its jobs and identified new potential areas of inquiry. A personal example is my 1991 physiological model of marathon running performance (Joyner, 1991). The ‘optimal’ time my model predicted was substantially faster than the world record in the early 1990s. Thus, my model raised questions about what was not known, especially about running economy. As the world record fell, my model morphed into a prediction and led to successful efforts to improve running economy via shoe technology and drafting tactics that have contributed to numerous world records (Senefeld et al., 2021).

## 5 BACK TO THE FURST AND GONZÁLEZ‐ALONSO MODEL

What Furst and González‐Alonso have done is to integrate what is known about the cardiovascular system in a different way, using a different perspective. The question is not whether their model is ‘right’; the question is what does it explain, what does it fail to explain and, most importantly, what testable questions does it frame for the rest of the world to explore?

## AUTHOR CONTRIBUTIONS

Sole author.

## CONFLICT OF INTEREST

The authors have no competing interests.

## FUNDING INFORMATION

There was no funding provided for this viewpoint article.
